# Randomized phase II trial of S‐1 plus cisplatin or docetaxel plus cisplatin with concurrent thoracic radiotherapy for inoperable stage III non‐small cell lung cancer

**DOI:** 10.1002/cam4.3641

**Published:** 2020-12-14

**Authors:** Tsuneo Shimokawa, Kazuhiko Yamada, Hiroshi Tanaka, Kaoru Kubota, Yuichi Takiguchi, Kazuma Kishi, Haruhiro Saito, Yukio Hosomi, Terufumi Kato, Daijiro Harada, Sakiko Otani, Takashi Kasai, Yoichi Nakamura, Toshihiro Misumi, Takeharu Yamanaka, Hiroaki Okamoto

**Affiliations:** ^1^ Department of Respiratory Medicine Yokohama Municipal Citizen’s Hospital Kanagawa Japan; ^2^ Department of Respiratory Medicine Shin Koga Hospital Fukuoka Japan; ^3^ Department of Internal Medicine Niigata Cancer Center Hospital Niigata Japan; ^4^ Department of Pulmonary Medicine, Infection and Oncology Nippon Medical School Tokyo Japan; ^5^ Department of Medical Oncology Chiba University Chiba Japan; ^6^ Department of Respiratory Medicine Toho University Tokyo Japan; ^7^ Department of Thoracic Oncology Kanagawa Cancer Center Kanagawa Japan; ^8^ Department of Respiratory Medicine Tokyo Metropolitan Cancer and Infectious Diseases Center Komagome Hospital Tokyo Japan; ^9^ Department of Thoracic Oncology Shikoku Cancer Center Ehime Japan; ^10^ Division of Medical Oncology Kanazawa University Cancer Research Institute Ishikawa Japan; ^11^ Department of Respiratory Medicine Tochigi Cancer Center Tochigi Japan; ^12^ Department of Biostatistics Yokohama City University Kanagawa Japan

**Keywords:** chemoradiotherapy, cisplatin, docetaxel, locally advanced non‐small‐cell lung cancer, S‐1

## Abstract

Cisplatin‐based chemoradiotherapy is considered standard treatment for unresectable locally advanced non‐small‐cell lung cancer (LA‐NSCLC). This study examined two regimens of chemotherapy in concurrent chemoradiation. Eligible patients with unresectable, radically irradible LA‐NSCLC were randomized to either the SP (S‐1 and cisplatin) or DP (docetaxel and cisplatin) arms with concurrent thoracic radiotherapy of 60 Gy, comprising 2 Gy per daily fraction. The primary endpoint was the overall survival (OS) rate at 2 years (the 2‐year OS rate). From May 2011 to August 2014, 110 patients were enrolled. Of 106 eligible patients, the 2‐year OS rates were 79% (95% CI: 66%–88%) and 69% (95% CI: 55%–80%) the SP and DP arms, respectively. The median progression‐free survival was 11.6 months for the SP arm and 19.9 months for the DP arm, while the median survival time was 55.2 months for the SP arm and 50.8 months for the DP arm. Grade 3/4 leukopenia were more frequent in DP arm. The incidences of febrile neutropenia and pneumonitis tended to be higher in DP arm. There were no treatment‐related deaths in either arm. The primary endpoint was met in both arms. The SP arm as a future reference regimen will be chosen due to fewer toxicities and better OS.

## Graphical abstract

This trial evaluated two experimental regimens that combine chemotherapy with concurrent radiotherapy. The S‐1 and cisplatin arm as a future reference regimen will be chosen due to fewer toxicities and better overall survival.

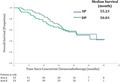

## 1 INTRODUCTION

Lung cancer is a leading cause of cancer deaths.^1^ Non‐small‐cell lung cancer (NSCLC) accounts for about 85% of all lung cancer ^2^ and about 25% of NSCLC is locally advanced disease (LA‐NSCLC).^3^ The standard treatment for patients with unresectable LA‐NSCLC is concurrent cisplatin‐based chemoradiotherapy.^4^, ^5^ However, the best concurrent chemoradiotherapy regimen has not yet been decided.^5^

During the last decade, it has been proved that the platinum‐based third‐generation chemotherapies contribute to the survival benefit of metastatic NSCLC.^6^, ^7^, ^8^ Furthermore, phase III studies of platinum‐based third‐generation chemotherapy with concurrent thoracic radiotherapy (TRT) for LA‐NSCLC provided promising survival benefit.^9^, ^10^ However, full‐dose chemotherapy with concurrent TRT using a combination of platinum and third‐generation agents is considered to be highly toxic. Therefore, for reduction in toxicity, weekly split chemotherapy has frequently been used in chemoradiotherapy with a combination of platinum and third‐generation agents. Because controlling distant metastases is important in curable lung cancer, it is necessary to increase the effect of chemotherapy to prevent recurrence of distant metastases.

In metastatic NSCLC, chemotherapy with cisplatin and docetaxel (DP) is one of the most effective regimens.^11^ In a phase I study, concurrent chemoradiotherapy with conventional and non‐split administration of DP is a tolerable and effective regimen in patients with LA‐NSCLC.^12^

S‐1 is a new oral fluoropyrimidine agent designed to increase anticancer effect and reduce gastrointestinal toxicity. It has promising in NSCLC.^13^, ^14^, ^15^, ^16^ The cisplatin and S‐1 combination chemotherapy (SP) in metastatic NSCLC proved to be a promising regimen in a phase III trial.^17^ In several phase II trials, concurrent chemoradiotherapy with SP was a promising treatment in patients with LA‐NSCLC.^18^, ^19^, ^20^, ^21^

We, the Thoracic Oncology Research Group (TORG), conducted a randomized phase II trial to compare these two treatment regimens and to demonstrate a standard treatment for patients with LA‐NSCLC.

## 2 MATERIALS AND METHODS

### 2.1 Patient Selection

Patients with histopathologically proven NSCLC with unresectable locally advanced stage were eligible. Eligible patients were also required to meet the following: no history of chemotherapy, TRT or surgical operation and could receive radiotherapy treatment according to the protocol (V20 under 35%). Other eligibility requirements included age of 20–74 years, Eastern Cooperative Oncology Group performance score (PS) of 0–1, and proper organ function.

To determine the stage, all patients received chest X‐ray (CXR), chest and abdomen computed tomography (CT), and either head CT or head magnetic resonance imaging (MRI). A bone scan or positron emission tomography (PET) as a bone metastasis test was also performed on all patients. The staging was decided based on the 7th Edition of the TNM classification.

All patients provided written informed consent before enrollment in this study. The protocol was designed in accordance with the Declaration of Helsinki and ethical guidelines for clinical research and was approved by the institutional review boards at all participating institutions.

### 2.2 Treatment protocol

Enrolled patients were randomly assigned to the SP arm or the DP arm at the TORG data center. Minimization was used for randomization and stratification factors were stage (IIIA or IIIB), gender (male or female), histopathological type (adenocarcinoma or non‐adenocarcinoma), and institution. Treatment consisted of concurrent chemoradiotherapy and subsequent consolidation chemotherapy. In the SP arm, patients took oral S‐1 twice a day after breakfast and supper on days 1–14 and cisplatin (60 mg/m^2^) as an intravenous administration on day 1. The dose of S‐1 was decided according to body surface area (BSA) as follows: BSA <1.25 m^2^, 80 mg per day; BSA 1.25 m^2^ to <1.50 m^2^, 100 mg per day; and BSA 1.5 m^2^ or higher, 120 mg per day. Chemotherapy was repeated twice at 4‐week intervals, concurrently with TRT. In the DP arm, patients were administrated docetaxel 50 mg/m^2^ and cisplatin 80 mg/m^2^ on day 1. Chemotherapy was repeated twice at 4‐week intervals, concurrently with TRT. In both groups, two more cycles of consolidation therapy were given 2 to 6 weeks after the completion of the concurrent chemoradiotherapy. In consolidation phase, chemotherapy was given every 3 weeks.

All patients were treated with 6–10 MV linear accelerator photon beam from day 1. The primary tumor and involved nodular disease received 60 Gy in fractions of 2 Gy over 6 weeks. In this protocol, a system scheduled for three‐dimensional (3‐D) treatment was basically acquired and 40 Gy of prophylactic mediastinal irradiation was administered. The initial 40 Gy/20 fractions were delivered to the clinical target volume 1 (CTV1) and the final 20 Gy/10 fractions to a reduced volume defined as clinical target volume 2 (CTV2). CTV1 included the area of the primary tumor, ipsilateral hilum, and mediastinal nodal areas from the paratracheal to subcarinal lymph nodes. The supraclavicular areas were not treated routinely but were treated when the supraclavicular nodes were involved. CTV2 contained only the primary tumor and the involved lymph nodes, with a margin of 0.5–1 cm.

TRT was suspended at the onset of grade 4 hematologic toxicity, more than grade 3 esophagitis or dermatitis, fever of 38 °C, more than grade 1 pneumonitis, or a decrease in the partial pressure of arterial oxygen of 10 Torr or more and more than grade 2 hypoxia. Patients were withdrawn from this study if a rest period of 2 weeks or more was required.

### 2.3 Evaluation of response and toxicities

All patients who received protocol treatment were evaluated for efficacy and safety. CXR, blood counts, and blood biochemical tests were performed weekly during the treatment period. Chest CT was taken every 1–2 months during the treatment period. After the conclusion of treatment, chest CT was performed every 6 months and other imaging tests were performed when recurrence was suspected. Responses were evaluated according to the Response Evaluation Criteria in Solid Tumor, version 1.1. Adverse events were evaluated according to the Common Terminology Criteria for Adverse Events (v4.0). Overall survival (OS) was defined as the time from registration until death from any cause. Progression‐free survival (PFS) was defined as the time between enrollment and disease progression, death, or last known follow‐up.

### 2.4 Statistical analysis

The full analysis set included all patients who received the protocol treatment at least once, were observed for survival, and did not violate the eligibility criteria. The safety analysis set included all patients who received at least one protocol treatment. The primary endpoint was the comparison of 2‐year OS rates between the SP and DP arms. This trial was to test the null hypothesis that the exact 2‐year OS rate was more than or equal to a threshold of 50% versus the alternative hypothesis that the exact 2‐year OS rate was ≤65%. In this design, the one‐sided α was 0.05. The two‐sided confidence interval (CI) of 90% for the 2‐year OS rate was used to verify the null hypothesis. The CI was calculated using Greenwood's formula. The patient assignment and follow‐up periods were 2 years. Given the possibility of variance inflation due to censoring, the sample size was set at 110. Baseline characteristics were compared among the treatment groups using Kruskal–Wallis and Fisher's exact tests for continuous and discrete variables, respectively. The rates of specific toxicities and treatment delivery were compared between the groups using Fisher's exact tests. Survival curves were estimated by the Kaplan–Meier method. All statistical analyses were performed using SAS version 9.4. We also evaluated OS, PFS, treatment completion rate, and safety as secondary endpoints.

## 3 RESULTS

### 3.1 Patient characteristics

**FIGURE 1:**
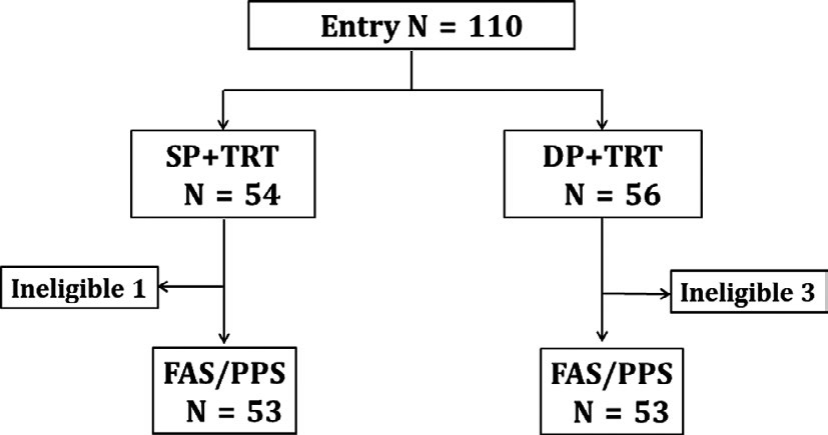
CONSORT diagram. SP: S‐1 and cisplatin, DP: docetaxel and cisplatin, TRT: thoracic radiotherapy, FAS: full analysis set, PPS: per‐protocol set

From May 2011 to August 2014, a total of 110 patients were registered for the study, and 54 and 56 patients were assigned to the SP and the DP arm, respectively, as shown in Figure 1. We measured efficacy and toxicity in 106 of the 110 patients. One case in the SP arm became ineligible because of metastatic lung cancer at re‐staging. In the DP arm, one patient each became ineligible because of impossibility of radiation according to protocol, agreement withdrawal, and rapid deterioration, respectively. The patient characteristics are listed in Table 1. There were no conspicuous differences in patient characteristics between the two arms.

**TABLE 1:** Patient characteristic.

		SP: *N* = 53	DP: *N* = 53
Gender	Male	42	41
	Female	11	12
Age (years)	Median (range)	63 (42–74)	66 (42–74)
Pathology	Adenocarcinoma	32	30
	Squamous cell carcinoma	14	11
	NOS	6	12
	LCNEC	1	0
Stage	IIIA	30	29
	IIIB	23	24
PS	0	32	27
	1	21	26
Smoking history	Yes	42	45
	No	11	8
EGFR mutation	Positive	7	9
	Wild‐type	29	32
	Unknown	17	12

Abbreviations: DP, docetaxel plus cisplatin; LCNEC, large‐cell neuroendocrine carcinoma; NOS, not otherwise specified; PS, performance status; SP, S‐1 plus cisplatin.

### 3.2 Treatment administration

The delivered dose intensities were the same (100%) for both arms during concurrent and consolidation treatment. Most patients completed TRT at 60 Gy (Table 2).

**TABLE 2:** Treatment delivery of drugs and radiation

		SP: *N* = 53	DP: *N* = 53
Chemotherapy (cycles)	1	5	1
	2	3	6
	3	3	4
	4	42 (79%)	42 (79%)
Relative dose intensity (median)		CDDP: 100% S−1: 100%	CDDP: 100% Docetaxel: 100%
Radiotherapy (Gy)	42	0	1
	59	1	0
	60	52 (98%)	52 (98%)

Abbreviations: CDDP, cisplatin; DP, docetaxel plus cisplatin; SP, S‐1 plus cisplatin.

### 3.3 Efficacy

In the SP arm, 38 patients responded to treatment (71.7%; 95% CI, 57.7% to 83.2%). In the DP arm, 36 patients responded to treatment (67.9%; 95% CI, 53.7% to 80.1%) (Table 3).

**TABLE 3:** Tumor response

		SP: *N* = 53	DP: *N* = 53
Best Response	CR	1	3
	PR	37	33
	SD	14	13
	PD	1	4
RR (%)	CR+PR [95%CI]	38 (71.7%) [57.7–83.2]	36 (67.9%) [53.7–80.1]
DCR (%)	CR+PR+SD [95%CI]	52 (98.1%) [89.9–100]	49 (92.5%) [81.8–97.9]

Abbreviations: CR, complete response; DCR, disease control rate; DP, docetaxel plus cisplatin; PD, progressive disease; PR, partial response; RR, response rate; SD, stable disease; SP, S‐1 plus cisplatin.

**FIGURE 2:**
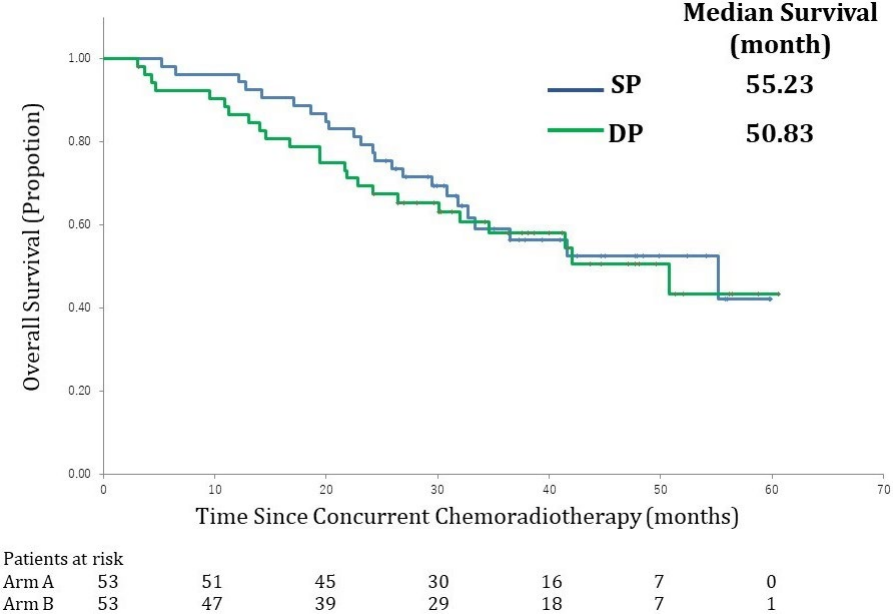
Survival curves. Kaplan–Meier estimates of overall survival (OS) in the full analysis set (FAS). OS was measured from the date of random assignment to the date of death for any cause. At the cutoff date for data inclusion in the analysis, if a patient had not died, the OS was censored at the last date they were known to be alive. SP arm: S‐1 plus cisplatin plus thoracic radiotherapy, DP arm: docetaxel plus cisplatin plus thoracic radiotherapy

**FIGURE 3:**
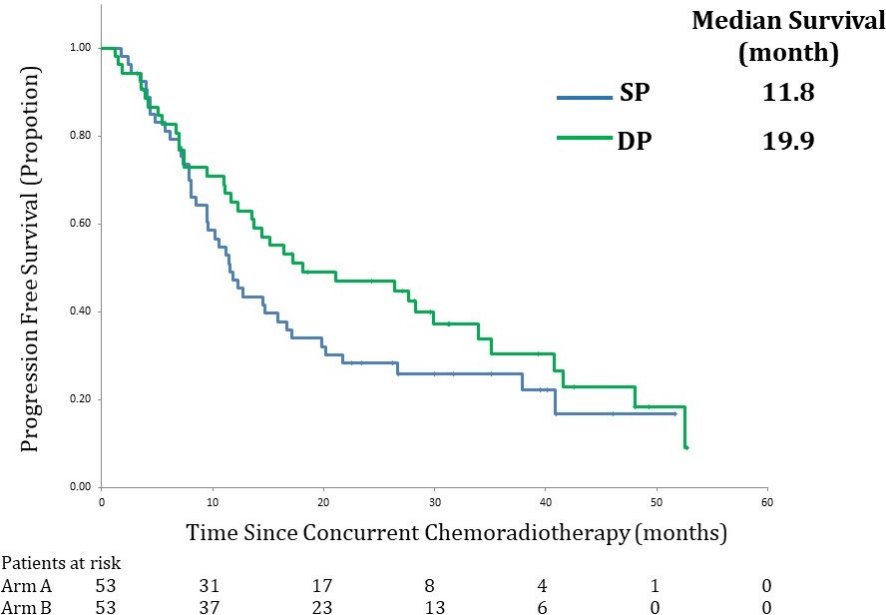
Survival curves. Kaplan–Meier estimates of progression free survival (PFS) in the full analysis set (FAS). PFS was defined as the time between enrollment and disease progression, death or last known follow‐up. SP arm: S‐1 plus cisplatin plus thoracic radiotherapy, DP arm: docetaxel plus cisplatin plus thoracic radiotherap.

Table 4 and Figures 2 and 3 show the OS and PFS for both arms. The 2‐year OS rate, a primary endpoint, in the SP arm was 79% (90% CI, 68% to 87%). The corresponding value was 69% (90% CI, 57% to 78%) in the DP arm. The primary endpoint was met in both arms as the lower limit level of the 90% CI exceeded the threshold of 50%.

**TABLE 4:** Survival

	SP: *N* = 53	DP: *N* = 53
Median PFS (months) [95% CI]	11.8 [9.5–17.1]	19.9 [12.3–29.9]
2‐year PFS (%) [95% CI] [90% CI]	30.2 [18.5–42.7] [20.3–40.7]	44.3 [30.6–57.1] [32.8–55.2]
5‐year PFS (%) [95% CI]	23.2 [12.6–35.8]	23.6 [12.3–36.9]
Median OS (months) [95% CI]	55.2 [32.7‐NR]	50.8 [30.1‐NR]
2‐year OS (%) [95% CI] [90% CI]	79.2 [65.7–87.9] [68.2–86.8]	69.3 [54.8–79.9] [57.4–78.4]
5‐year OS (%) [95% CI]	48.8 [33.0–62.8]	42.3 [23.9–59.5]

Abbreviations: DP, docetaxel plus cisplatin; OS, overall survival; PFS, progression free survival; SP, S‐1 plus cisplatin.

The median survival time (MST) in the SP arm was 55.2 months (95% CI, 32.7 to NR months) compared to 50.8 months (95% CI, 30.1 to NR months) in the DP arm.

The median PFS in the SP and DP arms were 11.8 (95%CI, 9.5 to 17.1 months) and 19.9 (95%CI, 12.3 to 29.9 months), respectively.

### 3.4 Survival recurrence patterns and subsequent treatment

In the SP arm, NSCLC recurred in a local site in 13 (24.5%), in a distant site in 18 (34.0%), and in both sites in 10 (18.9%) of 53 patients, compared to 10 (18.9%), 14 (26.4%), and 11 (20.8%) of 53, respectively, in the DP arm. The incidence of recurrence due to brain metastasis alone was 17.0% in the SP arm and 15.1% in the DP arm.

Subsequent treatment was administered in 73.6% and 54.7% of patients with recurrent disease in the SP and DP arms, respectively. The treatment regimens were as follows: chemotherapy (87.2%), radiotherapy (10.3%), and operation (2.5%) in the SP arm, and 72.4%, 17.2%, and 10.3% in the DP arm (data not shown). The numbers of posttreatment in patients with disease progression were shown in Table 5. Platinum‐based combination, cytotoxic agent monotherapy, bevacizumab with or without other agents, epidermal growth factor receptor (EGFR)/anaplastic lymphoma kinase (ALK)‐tyrosine kinase inhibitors (TKIs) and immune‐checkpoint inhibitors are 13 (24.5%), 24 (45.3%), 6 (11.3%), 13 (24.5%), and 6 (11.3%) in the SP arm, and 8 (15.1%), 20 (37.7%), 4 (7.5%), 7 (13.2%), and 3 (5.7%) in the DP arm.

**TABLE 5:** Poststudy chemotherapy in patients with disease progression

	Arm A (SP) (n = 37)	Arm B (DP) (n = 28)
Subsequent chemotherapy		
Platinum doublet	13 (24.5%)	8 (15.1%)
Single cytotoxic agents	24 (45.3%)	20 (37.7%)
Bev with or without other agents	6 (11.3%)	4 (7.5%)
TKI (EGFR, ALK)	13 (24.5%)	7 (13.2)
Immune‐checkpoint inhibitors	6 (11.3%)	3 (5.7%)
Others	5 (9.4%)	1 (1.9%)

Abbreviations: ALK, *anaplastic lymphoma kinase*; Bev, bevacizumab; EGFR, epidermal growth factor receptor; TKI, tyrosine kinase inhibitor

### 3.5 Toxicities

Treatment‐related toxicities in both arms are listed in Table 6. Grade 3 or greater leukocytopenia occurred significantly more frequently in the DP arm than in those in the SP arm. Febrile neutropenia tended to be higher in the DP arm.

**TABLE 6:** Adverse events

	SP: *N* = 53		DP: *N* = 53		Fisher's test *p*‐value
	All Grade	Grade 3–4	All Grade	Grade 3–4	Grade 3–4
Leukocytes	48	18	51	33	0.0031
Neutrophils	44	15	48	30	0.0028
Hemoglobin	48	5	52	7	0.3803
Platelets	44	0	32	3	0.1214
Anorexia	37	5	45	10	0.1324
Nausea	34	1	41	3	0.3089
Vomiting	7	0	12	1	0.5000
Diarrhea	14	2	19	2	0.6911
Constipation	31	1	28	0	0.5000
Fatigue	10	1	17	1	0.7524
Febrile neutropenia	1	1	6	6	0.0563
Lymphopenia	5	2	6	5	0.2185
Lung infection	2	2	4	3	0.5000
Esophagitis	26	2	23	2	0.6911
Pneumonitis	7	0	10	4	0.0589
Hyponatremia	5	2	7	1	0.5000

Abbreviations: DP, docetaxel plus cisplatin; SP, S‐1 plus cisplatin.

The incidence of radiation esophagitis (grade 3 or greater) was the same in both arms (3.8%). Radiation pneumonitis tended to be more severe in the DP arm. There was no grade 5 toxicity in either arm.

## 4 DISCUSSION

We carried out to compare S‐1 and cisplatin plus TRT to docetaxel and cisplatin plus TRT for LA‐NSCLC. Randomized phase III studies comparing third‐generation and second‐generation regimens combined with concurrent TRT showed that third‐generation regimens contribute to prolonging survival.^9^, ^10^ According to these findings, weekly carboplatin and paclitaxel with TRT or split docetaxel and cisplatin with TRT have become the commonly used regimens in Japan.

The present trial used the 2‐year survival rate as primary endpoint because judgment is difficult for response rates or the PFS of chemoradiotherapy treatment. Both SP and DP arms showed good outcomes in the present study. Both arms met the primary endpoint. Furthermore, to our knowledge, no other study has reported an MST of more than 50 months.

In the present study, PFS tended to be better in the DP arm, while OS tended to be better in the SP arm. One explanation for this discrepancy may be that subsequent treatment was performed less often in the DP (54.7%) than in the SP arm (73.6%) probably due to worse PS at the time of recurrence. Nonhematological toxicities tended to be higher in DP arm. Although late toxicities were not available in the CRF, more irreversible damages might possibly occur in the DP arm. Therefore, poststudy treatments were less frequently administered in the DP (54.7%) arm than the SP arm (73.6%) and consequently longer PFS of the DP arm could not translate into OS. The sites of relapse were comparable in both arms. However, the PS at the time of recurrence was not reported in the case report form (CRF) in this trial.

EGFR‐TKIs could also contribute to long‐term survival. EGFR mutation screening was not mandatory in this study. The rates of unknown EGFR mutations were 32.1% and 22.6% in the SP and DP arms, respectively. This high rate is one limitation of the present study. The higher rate of TKI administration in the SP arm may have contributed to the prolongation of OS in the SP arm. Immune‐checkpoint inhibitors as subsequent treatment were not examined at this time.

In this study, toxicities were very mild in the SP arm. Above all, the incidence of grade 3 or worse neutropenia in this arm was especially lower than that not only in the DP arm of this study, but also in other past study regimens. The incidence of radiation pneumonitis (grade 3 or greater) in the SP arm was 0%.

Recently, durvalumab, an anti‐PD‐L1 antibody, has been shown to be a promising agent for the consolidation phase.^22^, ^23^ Durvalumab will become generally used as maintenance therapy for LA‐NSCLC after induction chemoradiotherapy. However, durvalumab was approved during the study period and was not used in the present study. This point is another limitation. Despite this limitation, the study regimen is worth considering as an induction phase chemotherapy regimen. Preclinical data suggest that chemoradiotherapy may up‐regulate PD‐L1 expression in tumor cells, and the phase 3 study proved that PD‐L1 blockade can help restore systemic and long‐term immune response after chemoradiotherapy. It should be also noted that the potential mechanisms of SP regimen driving the interaction between immunotherapy and chemoradiotherapy are not well understood and warrants further investigation.

In conclusion, these results on the efficacy and toxicity indicate that S‐1 plus cisplatin with concurrent TRT will be a future reference regimen. This significant result requires examination in a future clinical trial including immune‐checkpoint inhibitors.

## 5 CLINICAL TRIAL REGISTRATION

This trial is registered at the University Hospital Medical Information Network (UMIN) Clinical Trial Registry (UMIN 000005993).

## AUTHOR CONTRIBUTIONS

Tsuneo Shimokawa wrote the manuscript. Tsuneo Shimokawa, Kazuhiko Yamada, Hiroshi Tanaka, Kaoru Kubota, Yuichi Takiguchi, Kazuma Kishi, Haruhiro Saito, Yukio Hosomi, Terufumi Kato, Daijiro Harada, Sakiko Otani, Takashi Kasai, Yoichi Nakamura, Toshihiro Misumi, Takeharu Yamanaka, and Hiroaki Okamoto conceptualized this study and revised the manuscript. Tsuneo Shimokawa, Kazuhiko Yamada, Hiroshi Tanaka, Kaoru Kubota, Yuichi Takiguchi, Kazuma Kishi, Haruhiro Saito, Yukio Hosomi, Terufumi Kato, Daijiro Harada, Sakiko Otani, Takashi Kasai, Yoichi Nakamura, and Hiroaki Okamoto looked after patients and collected the data. Toshihiro Misumi and Takeharu Yamanaka analyzed the data.

## Funding information

None declared.
